# Androgen Deprivation Induces Transcriptional Reprogramming in Prostate Cancer Cells to Develop Stem Cell-Like Characteristics

**DOI:** 10.3390/ijms21249568

**Published:** 2020-12-16

**Authors:** Shiv Verma, Eswar Shankar, F. Naz Cemre Kalayci, Amrita Mukunda, Malek Alassfar, Vaibhav Singh, E. Ricky Chan, Gregory T. MacLennan, Sanjay Gupta

**Affiliations:** 1 Department of Urology, School of Medicine, Case Western Reserve University, Cleveland, OH 44106, USA; sxv304@case.edu (S.V.); exs334@case.edu (E.S.); mxa778@case.edu (M.A.); 2 The Urology Institute, University Hospitals Cleveland Medical Center, Cleveland, OH 44106, USA; 3 The Hacettepe University Medical Center, Hacettepe University, Sihhiye, Ankara 06100, Turkey; kalaycicemre@gmail.com; 4 Krieger School of Arts and Sciences, Johns Hopkins University, Baltimore, MD 21218, USA; amukund1@jhu.edu; 5 Department of Inflammation and Immunity, Cleveland Clinic Foundation, Cleveland, OH 44195, USA; singhv2@ccf.org; 6 Department of Population and Quantitative Health Sciences, Case Western Reserve University, Cleveland, OH 44106, USA; erc6@case.edu; 7 Department of Pathology, Case Western Reserve University, Cleveland, OH 44106, USA; gtm2@case.edu; 8 Department of Nutrition, Case Western Reserve University, Cleveland, OH 44106, USA; 9 Division of General Medical Sciences, Case Comprehensive Cancer Center, Cleveland, OH 44106, USA; 10 Department of Urology, Louis Stokes Cleveland Veterans Affairs Medical Center, Cleveland, OH 44106, USA

**Keywords:** antiandrogens, castrate resistant prostate cancer, cancer stem cells, enzalutamide resistance, transcriptional reprogramming

## Abstract

Enzalutamide, an antiandrogen, is approved for therapy of castration resistant prostate cancer. Clinical applications have shown that approximately 30% of patients acquire resistance after a short period of treatment. However, the molecular mechanisms underlying this resistance is not completely understood. To identify transcriptomic signatures associated with acquisition of drug resistance we profiled gene expression of paired enzalutamide sensitive and resistant human prostate cancer LNCaP (lymph node carcinoma of the prostate) and C4-2B cells. Overlapping genes differentially regulated in the enzalutamide resistant cells were ranked by Ingenuity Pathway Analysis and their functional validation was performed using ingenuity knowledge database followed by confirmation to correlate transcript with protein expression. Analysis revealed that genes associated with cancer stem cells, such as *POU5F1* (OCT4), *SOX2*, *NANOG*, *BMI1*, *BMP2*, *CD44*, *SOX9*, and *ALDH1* were markedly upregulated in enzalutamide resistant cells. Amongst the pathways enriched in the enzalutamide-resistant cells were those associated with RUNX2, hedgehog, integrin signaling, and molecules associated with elastic fibers. Further examination of a patient cohort undergoing ADT and its comparison with no-ADT group demonstrated high expression of POU5F1 (OCT4), ALDH1, and SOX2 in ADT specimens, suggesting that they may be clinically relevant therapeutic targets. Altogether, our approach exhibits the potential of integrative transcriptomic analyses to identify critical genes and pathways of antiandrogen resistance as a promising approach for designing novel therapeutic strategies to circumvent drug resistance.

## 1. Introduction

Androgen deprivation therapy is the standard treatment for advance-stage prostate cancer [1,2]. Recent success of second generation drugs targeting androgen receptor axis including enzalutamide and abiraterone acetate, which blocks intratumoral production of androgen, have been approved by the Food and Drug Administration for the treatment of castration-sensitive and castration-resistant prostate cancer patients [3,4,5,6]. These agents have significant impact on treatment patterns; however, the majority of patients develop resistance to these drugs, despite their initial effectiveness, which in turn reflects our limited understanding of the mechanisms [7,8]. The failure of androgen deprivation therapy is largely associated with tumor heterogeneity that results in the differentiation of distinct cell subpopulations across and within disease sites.

Cancer stem cells (CSCs) are subpopulations of cancer cells that share similar characteristics as normal stem or progenitor cells within the tumor, retain the capacity of self-renewal and multi-lineage differentiation to drive tumor growth and heterogeneity [9,10,11]. The embryonic origin of prostate might explain the presence of stem cells within the organ [12]. Emerging evidence support a critical role of prostate CSC-like cells in stimulating castrate resistant evolution to enzalutamide and abiraterone acetate treatment [13,14]. We have earlier demonstrated that both naïve and therapy-resistant prostate cancer cell population have distinct subpopulations of CD133+ progenitor cells exhibiting significant tumorigenic ability and drug resistance [15]. Studies have further demonstrated that the distinct microenvironment and metabolic reprograming of cancer cells regulate CSCs differentiation and acquisition of drug resistance [16,17]. At the molecular level, CSCs express genes, such as *ALDH1, POU5F1* (OCT4), *CD44, NANOG*, *SOX2,* and others that play an essential role in maintaining stem cell pluripotency required to reprogram the differentiated cells. These genes subsequently promote lineage plasticity of cancer cells to adapt and develop resistance to cancer therapies [18,19]. Therefore, in-depth understanding and identification of cancer stem cell differentiation genes is critical for establishing novel tumor diagnostic and therapeutic strategy. We focused our attention to understand the underlying gene network promoting enzalutamide resistance that might be helpful in better designing of new therapeutic strategies.

In the present study, we induced transcriptional reprogramming in androgen-responsive human prostate cancer LNCaP and C4-2B cells through long-term enzalutamide exposure to develop resistance. We hypothesize that androgen deprivation therapy alters the phenotype of cancer cells and differentiate to cancer stem cell-like characteristics. Here we performed Next-Gen sequencing using both cell lines and compared them with their parental enzalutamide-sensitive counterparts, which were simultaneously exposed to vehicle for the same time period. After transcriptomic analysis, we performed integrative exploration using Ingenuity Pathway Analysis and ingenuity knowledge database to identify differentially regulated gene networks and analyzed these large-scale linkages to identify subnetworks associated with acquired enzalutamide resistance. A subset of genes residing within significant network were identified and further validated employing qRT-PCR, Western blotting and immunohistochemistry using resistant cells and clinical specimens from patients who underwent androgen deprivation therapy. Taken together, our approach identified nodes of sub-networks that may be putative therapeutic targets demonstrating translational significance.

## 2. Results

**Figure 1:**
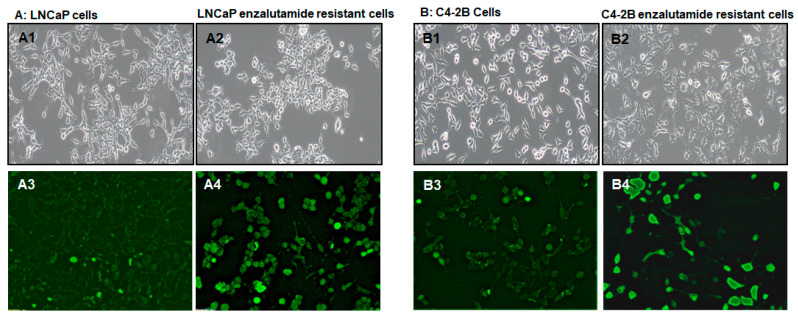
Self-renewal marker expression in pluripotent stem cells after enzalutamide exposure. The image shows differential staining of cancer stem-like cells in (**A**) LNCaP cells and (**B**) C4-2B cells with and without enzalutamide treatment. Morphological changes in parental cells (**A1**,**B1**) and enzalutamide treated cells (**A2**,**B2**). Staining with alkaline phosphatase, a stem cell marker in parental cells (**A3**,**B3**), and enzalutamide treated cells (**A4**,**B4**), respectively. Magnification 10×.

In this study, human prostate cancer LNCaP and C4-2B cells were used to generate enzalutamide resistant cells. These cell lines were cultured in medium containing 20 µM enzalutamide for six months followed by maintenance in 5 µM enzalutamide. Under the conditions, both LNCaP and C4-2B cells underwent morphological changes such as loss of cell-to-cell tight contact with scattered growth and temporary arm-like projections (Figure 1A1-2, B1-2). The growth curves for both cell lines demonstrate a marked difference in their sensitivity to enzalutamide treatment (Supplementary Materials Figure S1). To evaluate whether these phenotype changes in these cells were linked to the induction of stem-like characteristics and plasticity, we performed staining of live cells with fluorescence-tagged alkaline phosphatase. Alkaline phosphatase has emerged as a benchmark for identifying pluripotent stem cells [20,21]. Studies demonstrate that alkaline phosphatase is highly expressed in stem cells [22,23]. Live stain of LNCaP and C4-2B enzalutamide resistant cells demonstrate increased number and intensity of alkaline phosphatase positive cells, compared to parental cell lines. Higher intensity of alkaline phosphatase staining was noted in C4-2B enzalutamide resistant cells, compared to LNCaP enzalutamide resistant cells (Figure 1A3-4, B3-4).

**Figure 2:**
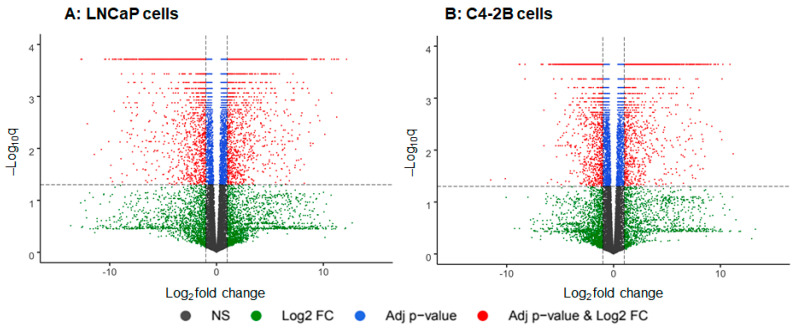
Volcano plots for differential gene expression analysis using R package-Enhanced volcano plot, (**A**) LNCaP cells, and (**B**) C4-2B cells with and without enzalutamide treatment. Each dot in the plot represents respective gene transcripts, red dots indicate the significantly up and downregulated genes with Log2 fold-change > 1 and adjusted *p*-value (FDR) < 0.05. Blue dot indicate genes that are significantly different with adjusted *p*-value (FDR) < 0.05 and does not meet fold change cut-off, while the grey and green color dots represent genes that did not pass the significant cut-off.

Next, we compared gene expression levels between enzalutamide resistant and parental cells (Figure 2). RNA-Seq analysis exhibited 35,504 expressed genes, 9409 differentially expressed genes (DEGs) were identified in LNCaP enzalutamide resistant cells, compared to parental counterpart (NCBI-GEO accession# GSE150807). In C4-2B enzalutamide resistant cells, 33,027 expressed genes and 7757 DEGs were identified, compared to parental cells (NCBI-GEO accession# GSE151083). Data visualization in the form of volcano plot display the relationship between magnitude of gene expression change (log2 fold-change; X-axis) and statistical significance of this change (−1og10 adjusted q value; Y-axis) in LNCaP and C4-2B enzalutamide resistant cells, compared with parental cell lines. Individual dots in volcano plot and scattered plot represent individual RNA gene transcripts in both cell lines (Figure 2A,B).

**Figure 3:**
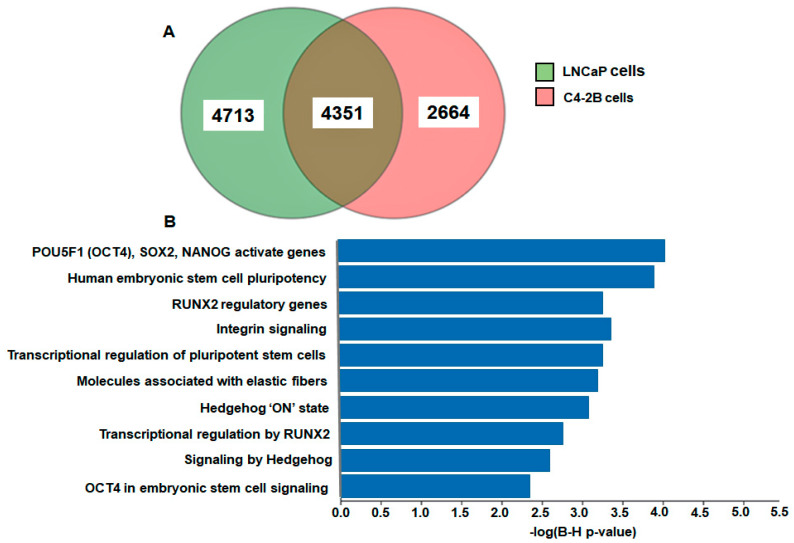
(**A**) Venn-diagram of differentially expressed genes (DEGs). The figure shows significantly elevated genes (*p*-value < 0.01) in LNCaP enzalutamide resistant cells (green) and C4-2B enzalutamide resistant cells (pink) compared to the parental cells. (**B**) Gene enrichment analysis. The DEGs were overlaid with ingenuity knowledge database of humans and to evaluate the definite overrepresented pathway(s), or to remove the chances of any randomness in data with reference to *p*-value, another statistical parameters of threshold value of 0.05 and Benjamin–Hochberg (B–H) was applied and represented in the form of bar graph with scale of gene and –log (B–H *p* value).

We next performed Ingenuity Pathway Analysis (IPA) and knowledge database on DEGs to investigate their biological relevance and pathway association. IPA analysis exhibited 4713 and 2664 differentially expressed genes (DEGs) in LNCaP and C4-2B enzalutamide resistant cells; whereas 4351 common DEGs in both cell lines (Figure 3A). The difference in the number of genes represented in the Venn-diagram (Figure 3) compared to Next Generation Sequencing (NGS) data (Figure 2) was due to the fact that some genes had infinite fold change values and were excluded. Moreover, the result of enrichment analysis analyzed through ingenuity knowledge database revealed *POU5F1* (*OCT4*), *SOX2*, and *NANOG* with the highest −log (B-H *p*-value), followed by human embryonic stem cell pluripotency, RUNX2 regulatory genes, integrin signaling, transcriptional regulation of pluripotent stem cells, molecules associated with elastic fibers, hedgehog “on” state, transcriptional regulation by RUNX2, signaling by hedgehog and, OCT4 in embryonic stem cell signaling, respectively (Figure 3B).

**Figure 4:**
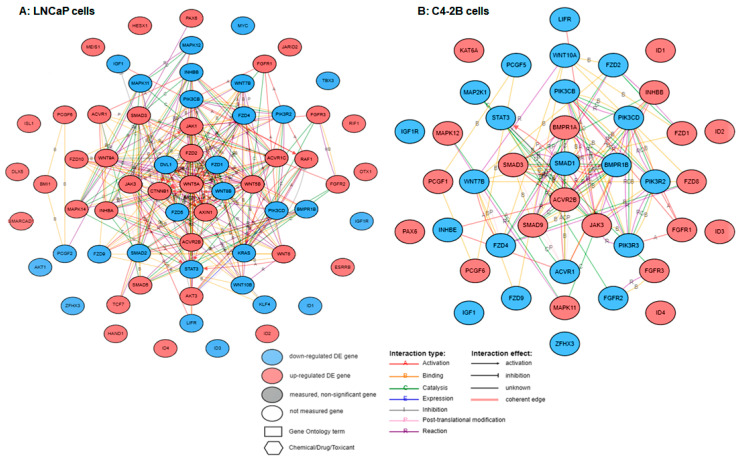
Gene network interaction of cancer stem cell signaling pathway in (**A**) LNCaP cells and (**B**) C4-2B cells with and without enzalutamide treatment. Red color indicates upregulated genes, blue indicates downregulated genes.

In the next set of experiments, we performed gene network analysis among DEGs of signaling pathway regulating stem cell pluripotency in both LNCaP and C4-2B enzalutamide resistant cells (Figure 4). The red color showed upregulation and blue color exhibited downregulation of differentially expressed genes in enzalutamide resistant cell lines. For instance, the expression of *ACVR1*, *ACVR1C*, *ACVR2B*, *AKT3*, *AXIN1*, *BMI1*, *CTNNB1*, *DLX5*, *ESRRB*, *FGFR1*, *FGFR2*, *FGFR3*, *FZD2*, *FZD10*, *HAND1*, *HESX1*, *ID2*, *ID4*, *INHBA*, *ISL1*, *JAK1*, *JAK3*, *JARID2*, *MAPK14*, *MEIS1*, *OTX1*, *PAX6*, *PCGF6*, *RAF1*, *RIF1*, *SMAD3*, *SMARCAD1*, *TCF7*, *WNT5A*, *WNT5B*, *WNT6*, and *WNT9A* were upregulated; whereas the expression of *AKT1*, *BMPR1B*, *DVL1*, *FZD1*, *FZD4*, *FZD5*, *FZD9*, *ID1*, *ID3*, *IGF1*, *IGF1R*, *INHBB*, *KLF4*, *KRAS*, *LIFR*, *MAPK11*, *MAPK12*, *MYC*, *PCGF2*, *PIK3CB*, *PIK3CD*, *PIK3R2*, *SMAD2*, *STAT3*, *TBX3*, *WNT10B*, *WNT7B*, *WNT8B*, and *ZFHX3* were downregulated in LNCaP enzalutamide resistant cells (Figure 4A). Similarly, the expression of *ACVR2B*, *BMPR1A*, *FGFR1*, *FGFR3*, *FZD1*, *FZD8*, *ID1*, *ID2*, *ID3*, *ID4*, *INHBB*, *JAK3*, *KAT6A*, *MAPK11*, *MAPK12*, *PAX6*, *PCGF1*, *PCGF6*, *SMAD3*, and *SMAD9* were upregulated; whereas *ACVR1*, *BMPR1B*, *FGFR2*, *FZD2*, *FZD4*, *FZD9*, *IGF1*, *IGF1R*, *INHBE*, *LIFR*, *MAP2K1*, *PCGF5*, *PIK3CB*, *PIK3CD*, *PIK3R2*, *PIK3R3*, *SMAD1*, *STAT3*, *WNT10A*, *WNT7B*, and *ZFHX3* are downregulated in C4-2B enzalutamide resistant cells (Figure 4B).

**Figure 5:**
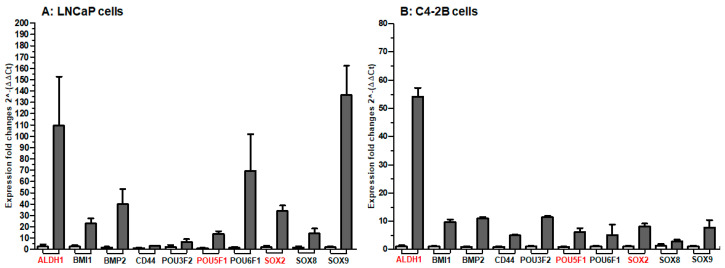
Real-time PCR analysis of stem cell marker genes in (**A**) LNCaP cells and (**B**) C4-2B cells with and without enzalutamide treatment. The abundance of transcripts ALDH1, BMI1, BMP2, CD44, POU3F2, POU5F1, POU6F1, SOX2, SOX8, and SOX9 were quantified in both resistant cells and their parental counterparts. GAPDH and actin were used as reference genes and set as baseline value to which all transcript levels were normalized. Y-axis in the bar represents expression fold change (2^-(ΔΔ*C*t) and X-axis shows the name of the transcript. Error bars refer to mean ± standard deviation for three technical and three biological replicates.

In order to validate our findings obtained from the RNA-Seq data, we selected a subset of 10 stem cell regulatory genes *viz*. *ALDH1*, *BMI1*, *BMP2*, *CD44*, *POU3F2*, *POU5F1*, *POU6F1*, *SOX2*, *SOX8*, and *SOX9* for their differential expression in LNCaP and C4-2B enzalutamide resistant cells in comparison with parental cells. In LNCaP enzalutamide resistant cells, *ALDH1*, *BMI1 BMP2*, *POU6F1, SOX2,* and *SOX9* were expressed in higher levels, compared with LNCaP cells at *p* value < 0.05. The fold change expression of *SOX9* (76.1), *ALDH1* (50), *POU6F1* (41.1), *BMP2* (26.6), *SOX2* (18.8), *BMI1* (10.5), *POU5F1* (9.3), *SOX8* (8.3), *POU3F2* (3.6), and *CD44* (3.1) was noted in the LNCaP enzalutamide resistant cells compared to LNCaP parental cells (Figure 5A). Similar trend of high gene expression was observed in C4-2B enzalutamide cells compared to C4-2B parental cells. The fold change expression of *ALDH1* (54), *POU3F2* (12.2), *BMP2* (11.5), *BMI1* (10), *SOX9* (8.7), *SOX2* (8.2), *POU5F1* (6.1), *POU6F1* (5.4), *CD44* (5), and *SOX8* (3.3) was noted in the enzalutamide resistant cells compared to C4-2B parental cells (Figure 5B).

**Figure 6:**
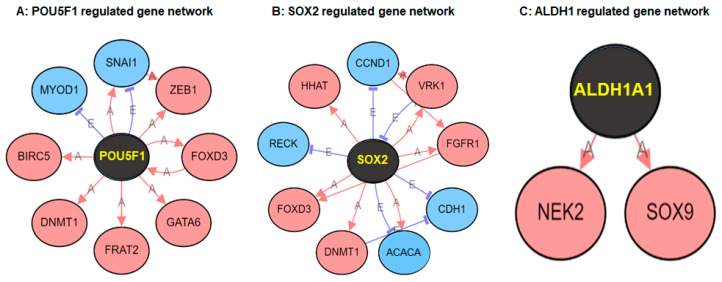
Differentially regulated genes (DEGs) as downstream target genes identified in LNCaP and C4-2B cells with and without enzalutamide treatment. (**A**) DEGs genes downstream of POU5F1 (OCT4), (**B**) DEGs genes downstream of SOX2, and (**C**) DEGs genes downstream of ALDH1, respectively. Red color indicates upregulated genes; blue indicates downregulated genes. Arrow head in red showed activation while blue head showed inhibition.

Based on the gene validation analysis, we identified a subset of differentially expressed genes *viz*. *ALDH1*, *POU5F1* (OCT4), and *SOX2* in both enzalutamide resistant cells. Next, we aimed to identify target genes based on Next-Gen sequencing curated databases. All nodes were sorted by degree, interaction types (activation, binding etc.) and interaction effect (activation, inhibition). Data showed the target genes, which were upregulated by *POU5F1,* included DNA methyltransferase 1 (*DNMT1*), Baculoviral IAP Repeat Containing 5 (*BIRC5*), FRAT regulator of WNT signaling pathway 2 (*FRAT2*), GATA Binding Protein 6 (*GATA6*), Forkhead Box D3 (*FOXD3*) and Zinc Finger E-Box Binding Homeobox 1 (*ZEB1*); whereas Myogenic Differentiation 1 (*MYOD1*), and Snail Family Transcriptional Repressor (*SNAI1*) were downregulated after binding with the homeodomain octamer motif (5′-ATTTGCAT-3′) of POU5F1 (Figure 6A). Similarly, *SOX2* upregulated target genes include hedgehog acyltransferase (*HHAT*), *FOXD3*, VRK Serine/Threonine Kinase 1 (*VRK1*), Fibroblast Growth Factor Receptor 1 (*FGFR1*), and *DNMT1* respectively, whereas *ALDH1A*, Chromodomain Helicase DNA Binding Protein 1(*CDH1*), Cyclin D1(*CCND1*), and Reversion inducing cysteine rich protein with kazal motifs (*RECK*) were downregulated after binding with SOX2 motif. (Figure 6B). Lastly, *ALDH1* upregulates 2 target genes that include NIMA Related Kinase 2 (*NEK2*) and *SOX9* (Figure 6C).

**Figure 7:**
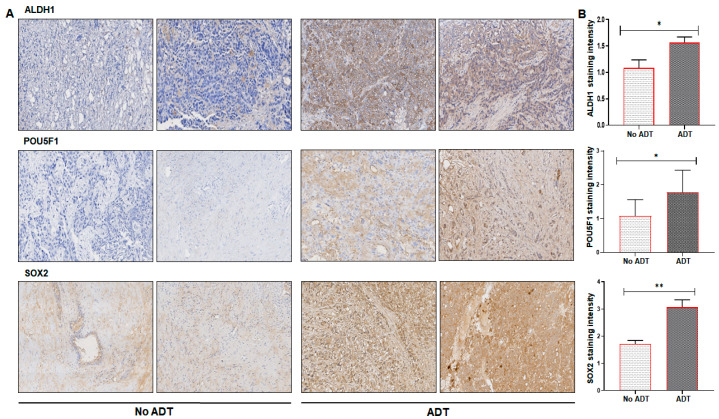
(**A**) Immunohistochemistry in paraffin-embedded tissue sections from patients who had undergone androgen deprivation therapy (*n* = 23) and specimens of grade-matched cancer tissues (*n* = 14) for protein expression of POU5F1 (OCT4), SOX2, and ALDH1 using anti-ALDH1, anti-POU5F1, and anti-SOX2 antibodies. (**B**) Statistical significance of immunohistochemistry was in lieu of the form of bar graph, error bars represent standard deviation * *p*< 0.05 and ** *p* < 0.001, compared to ADT versus no-ADT. Magnification 10×.

Next, we performed immunohistochemistry for ALDH1, POU5F1, and SOX2 in clinical specimens of the prostate from patients who had undergone androgen deprivation therapy and the tissues were obtained post-ADT treatment from the tumors. The grade-matched tumor samples, which did not received any adjuvant treatment, were included in the study, and served as controls. The ALDH1 expression was specifically detected in luminal cancer cells and exhibited broad variations, ranging from negative to focal, diffuse, or positive—albeit patchy and milder in samples with no-ADT, compared to the ADT specimens. Typically, ALDH1 expression was higher and more frequently expressed in tumor cells. Immunohistochemistry with POU5F1 antibody on specimens with no-ADT exhibited weak positive staining or no staining in luminal cancer cells, whereas positive cytoplasmic staining was noted in basal cells. In sharp contrast, specimens with ADT exhibited moderate POU5F1 staining patterns with strong nuclear presence in both the luminal and basal cells. IHC performed for SOX2 in no-ADT specimens exhibited a mixed basal and luminal epithelial cell staining in cancer with higher percentage of SOX2 positive cells, specifically in basal cells. A marked increase with uniform strong SOX2 expression was noted in specimens with ADT (Figure 7A). The total number of ALDH1, POU5F1, and SOX2 positive cells were quantified in both no-ADT and ADT specimens in 10–12 fields from each specimen, and compared using unpaired Student’s t-test (Figure 7B).

To further confirm whether antiandrogen treatment resulted in higher expression of stem cell markers, we performed Western blotting for AR, AR-v7, ALDH1, POU5F1, and SOX2 in LNCaP and C4-2B enzalutamide resistant cells and their parental counterparts. The AR protein expression were significantly decreased in both enzalutamide resistant cells whereas AR-v7 expression was increased in C4-2B enzalutamide resistant cells. A marked increase in the protein expression of ALDH1, POU5F1, and SOX2 were noted in both enzalutamide resistant cells, compared to their parental counterparts (Supplementary Materials Figure S2).

## 3. Discussion

In phase III clinical trials, the second-generation AR antagonist enzalutamide has been demonstrated to improve survival for patients with metastatic CRPC [24,25,26]. However, patients who initially respond to enzalutamide ultimately develop chemo-resistance and undergo disease progression [3,27]. Because of this, there is an urgent need to identify mechanisms of drug resistance. Approximately 30% of advanced-stage prostate cancer patients’ exhibit cellular plasticity and acquisition of altered phenotypes often associated with the loss of AR signaling and/or alteration in AR splice variants [28,29]. In this study, we used the IPA to identify mechanisms of enzalutamide resistance that could be developed as therapeutic drug target(s). The IPA analysis identified 4351 common subset of genes differentially expressed in both resistant cell lines. Our data demonstrate that the subset of overlapping genes is enriched with stem cell marker genes, and these show high levels of expression in both LNCaP and C4-2B enzalutamide-resistant cell lines. In particular, the LNCaP enzalutamide-resistant cell line exhibited stem cell marker genes associated with embryonic stem cells, mesenchymal stem cells, hematopoietic stem cells, and more neural stem cells (Supplementary Materials Figure S3A). The C4-2B enzalutamide-resistant cell lines showed higher percentage of embryonic stem cells, hematopoietic stem cells, mammary stem cells and mesenchymal stem cells (Supplementary Materials Figure S3B). These subsets of cell populations within the tumor change the microenvironment and may account for resistance against antiandrogens.

Next, we focused on pathway analysis using IPA knowledge database as this approach can identify genes implicated in specific signaling networks. Our study showed enriched signaling pathways that include hedgehog in ‘off’ and ‘on’ state, transcriptional regulation of RUNX2, molecules associated with elastic fibers, integrin signaling and transcriptional regulation of pluripotent stem cells. We observed enrichment of human embryonic stem cell pluripotency genes along with transcriptional regulation of pluripotent stem cells that might lead to activation of *POU5F1* (OCT4), *SOX2, CD44, NANOG,* and other genes associated with self-renewal and drug resistance of cancer cells. Among these signaling pathways, regulating stem cell pluripotency were involved in self-renewal, having potential to differentiate various cell types of the 3 germinal layer. These signaling pathways converge to activate the core transcription factors, such as OCT4, SOX2, NANOG, and others, which are involved in self-renewal of cells [30,31,32]. These transcription factors and their downstream target genes coordinate to promote self-renewal, pluripotency, and drug resistance (Supplementary Materials Figure S4).

Gene network analysis of signaling pathways that regulate pluripotency of stem cells showed upstream and downstream regulatory gene interactions [33,34]. The analysis showed molecules associated with WNT signaling pathway such as WNT5A, WNT6, WNT9A, and others, which include AKT3, FGFR1, FGFR2, FGFR3, BMI1 (upregulated in nodes of LNCaP enzalutamide resistant cells) that promote embryonic stem cell differentiation and self-renewal. Along with the above EP300, SMAD3, SMAD4, and others are additional subsets of gene regulatory network, which regulate the TGF-β signaling pathway. In C4-2B enzalutamide resistant cells, SMAD3, SMAD9, FGFR1, FGFR3, BMPR1A, and others were upregulated, acting in concert with the downstream molecules. Furthermore, the TGF-β signaling pathway activates BMP that binds with BMPR activating SMAD-like protein, along with SOX2 and OCT4 for their involvement in the differentiation, self-renewal, and drug resistance (Supplementary Materials Figure S5).

Several transcription factors have been shown to be involved in the initiation and maintenance of stemness in prostate cancer [35,36]. Examination of castration-resistant and hormone-naïve prostate cancer datasets demonstrate similar functionally validated genes expressed in post-androgen deprivation therapy specimens [32,37,38]. Several studies have shown that ALDH1 is expressed to different degrees in most human tumors [39,40]. In mantle cell lymphoma, a small population of CSCs expressing ALDH showed resistance to a wide range of chemotherapeutic agents [41]. Moreover, silencing of ALDH1A1 using nano-liposomal siRNA sensitized both taxane-and platinum resistant cell lines to chemotherapy in ovarian cancer [42]. High expression of ALDH1 at the transcript or protein level in tumors has been implicated in drug resistance and disease relapse [43,44]. A positive association between ALDH1 expression and poor prognosis was observed in core biopsies and tissue microarrays of prostate cancer patients [45]. Our study also demonstrates similar results with increase in ALDH1 levels in drug resistant cells and prostate tumors with ADT. Our analysis further identified the mitotic kinase NEK2 and stem cell transcription factor SOX9 as downstream targets of ALDH1, which might be involved in the development of multi-drug resistance in prostate cancer cells. Additional studies are required to validate these findings.

Elevated expression of POU5F1 (OCT4) both at the transcript and protein levels confers self-renewal of CSCs, and may lead towards enzalutamide resistance [46]. Likewise, high expression of OCT4 was observed in breast CSCs, and correlates with therapeutic resistance [47]. OCT4 regulates stem cell pluripotency and differentiation through regulation of DNMT1 [48,49]. OCT4 and NANOG upregulate DNMT1 through direct binding to its promoter to prevent the cells from reverting to an undifferentiated state [50]. Other OCT4 target genes including FRAT2, FOXD3, GATA6, and ZEB1 have been shown to play a significant role in pluripotency and self-renewal of CSCs [51,52]. Furthermore, increased expression of OCT4 target gene NAIP/BIRC5 has been associated with therapeutic resistance in various human cancers [53,54]. We found upregulation of these genes in enzalutamide resistant cell lines, which could function as determinants of multidrug resistance and tumor aggressiveness. These molecules may serve as potential new molecular targets to overcome enzalutamide resistance in prostate cancer.

Studies have shown that SOX2 overexpression leads to increase cell proliferation and promotes invasion, migration, and metastasis in various human cancers [55,56,57]. In prostate cancer, SOX2 has been shown to promote resistance to antiandrogen therapy by initiating lineage plasticity [58]. Studies reference SOX2 as an androgen repressed gene where AR binds the enhancer element within the SOX2 promoter to regulate its expression [28]. Aberrant SOX2 expression is noted, particularly in castration-resistant prostate cancer cells where ligand activation of AR promotes a decrease in SOX2 expression as a result of direct binding of the AR to the SOX2 cis-enhancer region [28,59]. In fact, SOX2 appears to promote castration resistance via mechanisms that do not involve the re-expression of embryonic stem cell SOX2-target genes but provides survival advantage through the loss of tumor suppressors including p53 and Rb genes [60,61]. In our study, we identified *DNMT1* and *FOXD3* as common subsets of genes upregulated by *POU5F1* (OCT4) and *SOX2*. Further studies, however, are required to confirm these findings.

## 4. Materials and Methods

### 4.1. Chemicals and Reagents

Enzalutamide (Cat# A10562) was purchased from Adooq Bioscience, Irvine, CA, USA, and all other reagents purchased were of GLP grade. Antibodies including Anti-AR (Cat# 5153), anti-AR-v7 (Cat# 19672), anti-POU5F1 (OCT4) (Cat# 2750S) were purchased from Cell Signaling Technologies, Beverly, MA, USA. The anti-ALDH1 (Cat# SC-166362), anti-SOX2 (Cat# SC-365823), anti-α-GAPDH (Cat# SC-47724), goat anti-mouse IgG-HRP (Cat# SC-2005), bovine anti-goat IgG-HRP (Cat# SC-2350), and goat anti-rabbit IgG-HRP (Cat# SC-2004) antibodies were purchased from Santa Cruz Biotechnology, Dallas, TX, USA.

### 4.2. Cell Culture

Human prostate cancer LNCaP and C4-2B cells were grown in RPMI 1640 (Cat# SH30027.01, GE Healthcare, Marlborough, MA, USA) supplemented with 10% fetal bovine serum, 50 U/mL penicillin and 50 µg/mL streptomycin in 100-mm tissue culture plates at 37 °C in a humidified atmosphere (5% CO_2_). These cells were used for generating enzalutamide resistant clones by exposing the cells to 20 µM enzalutamide for a minimum of six months and maintaining in media containing 5 µM enzalutamide. The absence of mycoplasma contamination was tested using PCR-based assay (Cat# MP0025; Sigma-Aldrich, St. Louis, MO, USA). The parental cells were maintained in the drug vehicle for the same time period and served as corresponding controls.

### 4.3. Alkaline Phosphatase Staining

LNCaP and C4-2B enzalutamide resistant and parental cells were stained for alkaline phosphatase activity as a measure of pluripotency using alkaline phosphatase live stain reagent (Cat# 14353, Thermo Fisher Scientific, Grand Island, NY, USA). The dye is a non-toxic, cell-permeable fluorescent substrate for alkaline phosphatase that diffuses out over the course of two hours. The cells were incubated with the substrate for 20–30 min and washed twice with DMEM/F-12 media to remove excess reagent. Following the final wash, fresh media was added and images were captured within 30–60 min after staining. Visualization of fluorescent-labeled cells were observed under fluorescent microscopy using a standard FITC filter.

### 4.4. Library Preparation and Next Generation Sequencing (NGS)

Total RNA was extracted from both LNCaP and C4-2B enzalutamide resistant and sensitive cells continuously exposed to enzalutamide (5 µM), using RNA RNeasy kit (Qiagen, Maryland, MD, USA). The total RNA integrity (RIN) was assessed using an RNA 6000 nanochip (Agilent Technologies, Santa Clara, CA, USA) on a Bioanalyzer 2100 (Agilent Technologies). Libraries were prepared using the Illumina TruSeq Stranded Total RNA Sample Preparation kit according to the manufacturer’s protocol. The 50 bp single-end sequencing was performed on pooled libraries using an Illumina HiSeq 2500 platform. Library preps and sequencing were completed by the Case Western Reserve University Genomics Core Facility.

### 4.5. NGS Data Analysis and Visualization

Sequencing reads generated from the Illumina platform were assessed for quality using FastQC. Illumina HiSeq 2500 reads were trimmed and clipped for quality control in TrimGalore v0.4.3 a wrapper script for cutAdapt and FastQC. Alignment of the data was performed using STAR Aligner v2.5.3 using the human reference genome GRCh38 and the GENCODE transcript annotation v25. Differential expression was determined using Cufflinks v2.2.1. Differentially expressed genes were identified using a multiple testing corrected *q*-value < 0.05. Mitochondrial chromosome and the non-chromosomal sequences were excluded from the analysis. The Next-Gen sequencing data of LNCaP and C4-2B enzalutamide resistant cells were submitted to NCBI-GEO having accession number GSE150807 and GSE151083, respectively.

### 4.6. Pathway and Gene Set Enrichment Analysis

Pathway analysis was performed using Ingenuity Pathway Analysis v 5.0 (IPA, Qiagen), the differentially expressed genes (DEGs) were imported into the IPA software and were subjected to functional annotations and regulatory network analysis. The DEGs were overlaid with ingenuity knowledge database of humans, and, to evaluate the definite overrepresented pathway(s), or to remove the chances of any randomness in data with reference to *p*-value, another statistical parameter of threshold value of 0.05 and Benjamin–Hochberg (B–H) was applied and represented in the form of bar graph, with scale of gene and −log (B–H *p* value).

### 4.7. Quantitative Real-Time PCR

Total RNA was isolated from enzalutamide resistant and respective parental cell lines, and RNA quality was analyzed using NanoDrop ND-1000 Spectrophotometer (NanoDrop, Wilmington, DC, USA). 1 µg total RNA was used for cDNA synthesis (Applied Biosystems™, Foster City, CA, USA) using High-Capacity cDNA Reverse Transcription Kit (Thermo Fisher, Waltham, MA). To quantify and amplify the gene oligonucleotides designed by Integrated DNA Technologies (Coralville, IA, USA) were used. The list of the genes probed are mentioned in Table 1, and GAPDH (NM_008084), and Actin (NM_007393) were used as internal control in the reaction. All reactions were performed in triplicate (three biological and three technical replicates) along with no template controls (NTC). The reaction for qRT was setup accordingly; 2.5 µl of SYBR green (Radiant™ SYBR Green low-ROX qPCR, Alkali Scientific, Fort Lauderdale, FL, USA) of 5× sample were added for a total 10 μL volume with thermal cycler program used started at 50 °C for 2 min then proceeded with 95 °C for 10 min for initial denaturing, followed by 40 cycles of 95 °C for 15 s, 60 °C for 40 s, and 72 °C for 35 s to collect cycle threshold (*C*t) values, along with dissociation curve cycle. The 2-ΔΔ*C*t method was used to calculate relative expression of each gene as previously described [62].

### 4.8. Western Blotting

The protein content in the cell lysates from LNCaP and C4-2B enzalutamide resistant cells and parental counterparts was determined using the DC Bio-Rad protein assay kit. The 40 μg of protein was resolved using 4–20% polyacrylamide gels (Novex, Carlsbad, CA, USA) and transferred to a nitrocellulose membrane. The blot was blocked in blocking buffer (5% nonfat dry milk/1% Tween 20; in 20 mM TBS, pH 7.6) for 2 h at room temperature, incubated with appropriate primary antibody for 2 h at room temperature or overnight at 4 °C, followed by incubation with the appropriate IgG secondary antibody conjugated to horseradish peroxidase (Amersham-Pharmacia, Piscataway, NJ, USA), and detected by ECL-chemiluminescence (Alkali Scientific Inc., Fort Lauderdale, FL, USA) and autoradiography using XAR-5 film (Eastman Kodak, Rochester, NY, USA).

### 4.9. Clinical ADT and Non-ADT Prostate Tissue Specimens

Specimens of post-surgical human prostate tissue from patients who had undergone androgen deprivation therapy were procured from the Tissue Procurement Facility at the University Hospitals Cleveland Medical Center. Consent was not prerequisite for these discarded tissues per hospital policies and the Institutional Review Board. Approval for this study was confirmed by the Institutional Review Board at the University Hospitals (STUDY20200324). Additional grade matched tumor tissue specimens were obtained from patients having undergone surgical procedures for prostatic disease without having received any adjuvant therapy.

### 4.10. Immunohistochemistry

Samples from patients undergone androgen deprivation therapy (*n* = 23) and specimens of grade-matched cancer tissues (*n* = 14) were sliced in 4 µm sections, de-paraffinized, rehydrated, and immersed in a target retrieval solution and blocked for endogenous peroxidase activity. Sections were permeabilized in TNB-BB (100 mM Tris, pH 7.5, 150 mM NaCl, 0.5% blocking agent, 0.3% Triton-X and 0.2% saponin) and incubated with anti-ALDH1, anti-POU5F1, and anti-SOX2 antibodies overnight at 4 °C, followed by incubation with biotinylated secondary antibody. The immunoreactive complexes were detected using diaminobenzidine substrate-chromogen on an inverted Olympus BX51 microscope. Images were acquired with Olympus MicroSuite^TM^ Five Software (Soft Imaging System, Lakewood, CO, USA) and the staining intensity was graded semi-quantitatively. Each specimen was assigned a score on a scale from 0 to 3 designated as 0 (no staining), 1 = weak staining, 2 = moderate staining, 3 = strong staining, reported as 0–3 staining score.

### 4.11. Statistical Analysis

Unpaired two-tailed student’s t-test was used to compare gene expression LNCaP and C4-2B enzalutamide resistant cells and parental counterparts and results were presented as mean ± SD, *p* value < 0.05 was considered statistically significant.

## 5. Conclusions

Our study demonstrates high expression of ALDH1, POU5F1 (OCT4), and SOX2 during androgen deprivation therapy maintain pluripotency and self-renewal through regulation of several target genes and gene encoding components of key signaling pathways. Further studies targeting CSCs and their key signaling pathways is a promising approach for designing novel therapeutic strategies to circumvent chemo-resistance in castration resistant prostate cancer.

## Figures and Tables

**Table 1:** List of primers.

Gene	Forward Primer (5′ to 3′)	Reverse Primer (5′ to 3′)
*ALDH1*	GTCAAACCAGCAGAGCAAAC	GGCCCATAACCAGGAACAATA
*BMI1*	ATCAGTCACCAGAGAGATGGA	GGGCTAGGCAAACAAGAAGA
*BMP2*	CAGCTGTAAGAGACACCCTTTG	GCATTCTCCGTGGCAGTAAA
*CD44*	GCAGGTATGGGTTCATAGAAGG	GGTGTTGGATGTGAGGATGT
*POU3F2*	CTGGAGAGCCATTTCCTCAAA	AAACCAAACTCTCACCACCTC
*POU5F1*	GGAGGAAGCTGACAACAATGA	CTCTCACTCGGTTCTCGATACT
*POU6F1*	CTCCACAGCACCACTCAATA	GGTTACAGTGAGGCGAGATT
*SOX2*	CGTTCATCGACGAGGCTAAG	CTTCTTCATGAGCGTCTTGGT
*SOX8*	GTGTCGCAGGTGCTCAA	TTCATGGGCCGCTTCAC
*SOX9*	TCTGGAGACTTCTGAACGAGAG	CGCGGCTGGTACTTGTAATC

## Supplementary Materials

Supplementary materials can be found at https://www.mdpi.com/1422-0067/21/24/9568/s1. Figure S1: (A) Sensitivity to enzalutamide treatment in LNCaP and C4-2B parental and enzalutamide resistant cells. (B) Methylene blue assay in LNCaP and C4-2B parental and enzalutamide resistant cells, Figure S2: Representative image of protein expression of AR, AR-v7, POU5F1 (OCT4), SOX2 and ALDH1 in LNCaP and C4-2B cells with and without enzalutamide treatment, Figure S3: *In-silico* stemness signatures for (A) LNCaP and (B) C4-2B enzalutamide resistant cell lines represented in radar chart, Figure S4: Signaling pathway regulating pluripotency of stem-cell leading to pluripotency maintenance, Figure S5: Human embryonic stem cell pluripotency signaling pathway.

## Abbreviations

ADT	Androgen deprivation therapy
ALDH	Aldehyde dehydrogenase
BIRC5	Baculoviral IAP Repeat Containing 5
BMI1	Polycomb complex protein
BMP2	Bone Morphogenetic Protein 2
CCND1	Cyclin D1
CDH1	Chromodomain Helicase DNA Binding Protein 1
CSCs	Cancer stem cells
DEGs	Differentially expressed genes
DNMT1	DNA methyltransferase 1
FGFR	Fibroblast Growth Factor Receptor
FOXD3	Forkhead Box D3
FRAT2	FRAT regulator of WNT signaling pathway 2
GATA6	GATA Binding Protein 6
HHAT	Hedgehog acyltransferase
LNCaP	Lymph node carcinoma of the prostate
MYOD1	Myogenic Differentiation 1
NEK2	NIMA Related Kinase 2
OCT4	Octamer-binding transcription factor 4
RECK	Reversion inducing cysteine rich protein with kazal motifs
RUNX2	Runt-related transcription factor 2
SNAI	Snail Family Transcriptional Repressor
VRK1	VRK Serine/Threonine Kinase 1
ZEB1	Zinc Finger E-Box Binding Homeobox 1
