# Supplementary material for: Androgen Deprivation Induces Transcriptional Reprogramming in Prostate Cancer Cells to Develop Stem Cell-Like Characteristics
Source: Int J Mol Sci. 2020 Dec 16;21(24):9568. doi: 10.3390/ijms21249568 (PMC7765584; doi:10.3390/ijms21249568)
Supplement: Supplementary file 1 [file ijms-21-09568-s001.pdf]

Supplemental Figure 1

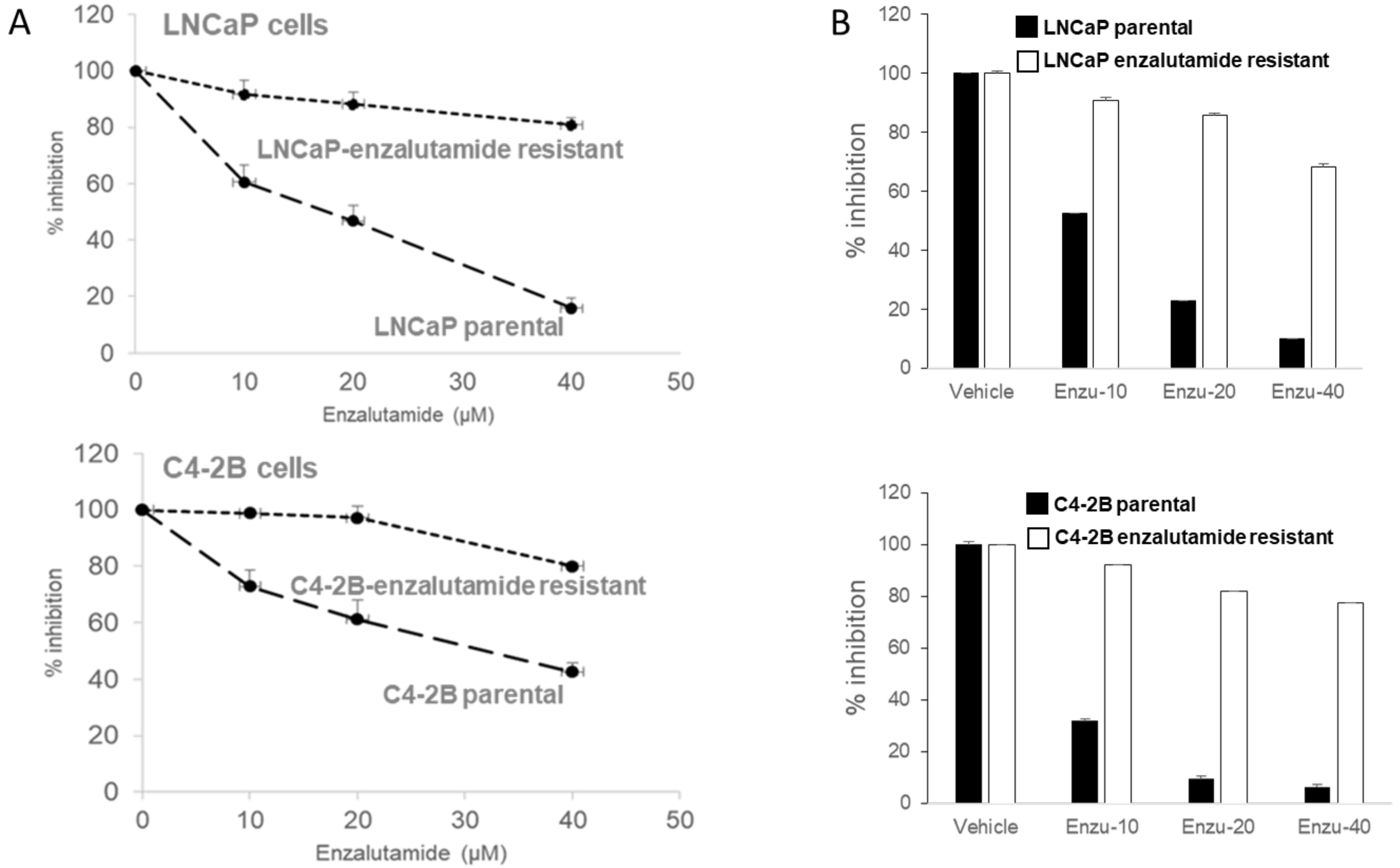

Figure S1. (A) Sensitivity to enzalutamide treatment in LNCaP and C4-2B parental and enzalutamide resistant cells. The cells were treated with increasing doses of enzalutamide (10-40 µM) in 0.1 % DMSO for 48 h. Cell viability was measured by MTT assay. **(B)** Methylene blue assay in LNCaP and C4-2B parental and enzalutamide resistant cells.

Supplemental Figure 2

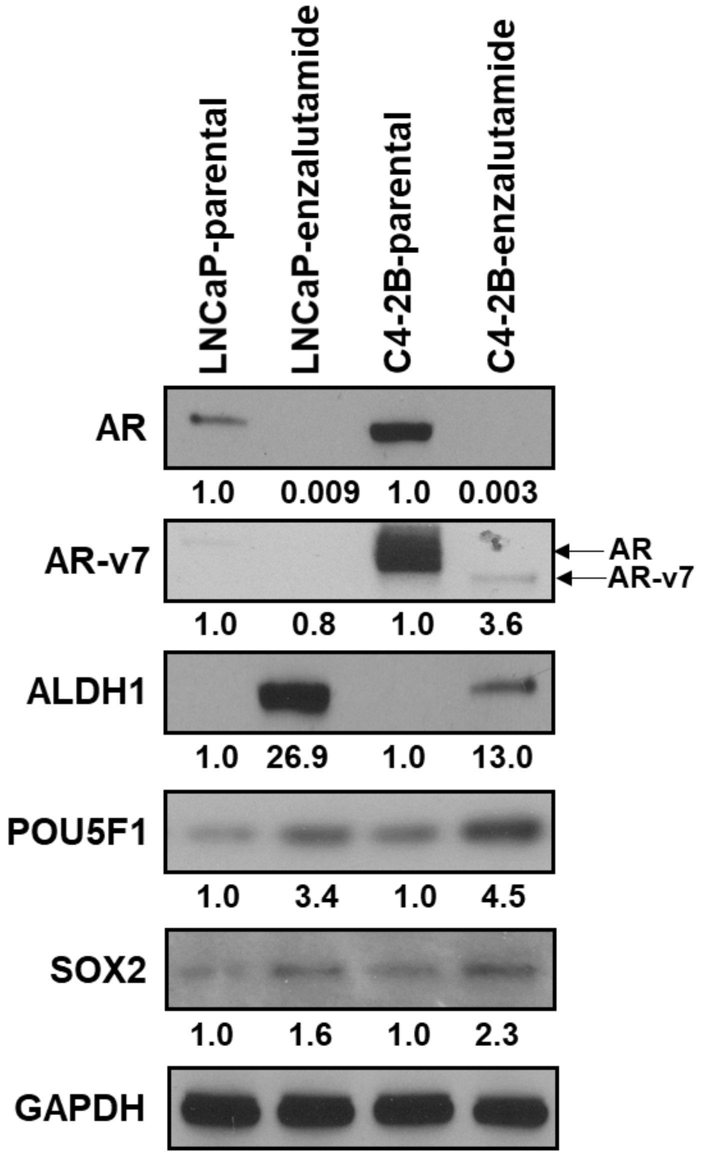

Figure S2. Representative image of protein expression of AR, AR-v7, POU5F1 (OCT4), SOX2 and ALDH1 in LNCaP and C4-2B cells with and without enzalutamide treatment. Densitometry data is provided below each blot using GAPDH as the loading control.

Supplemental Figure 3

A: LNCaP enzalutamide resistant cells **C42B-enzalutamide resistant cells**

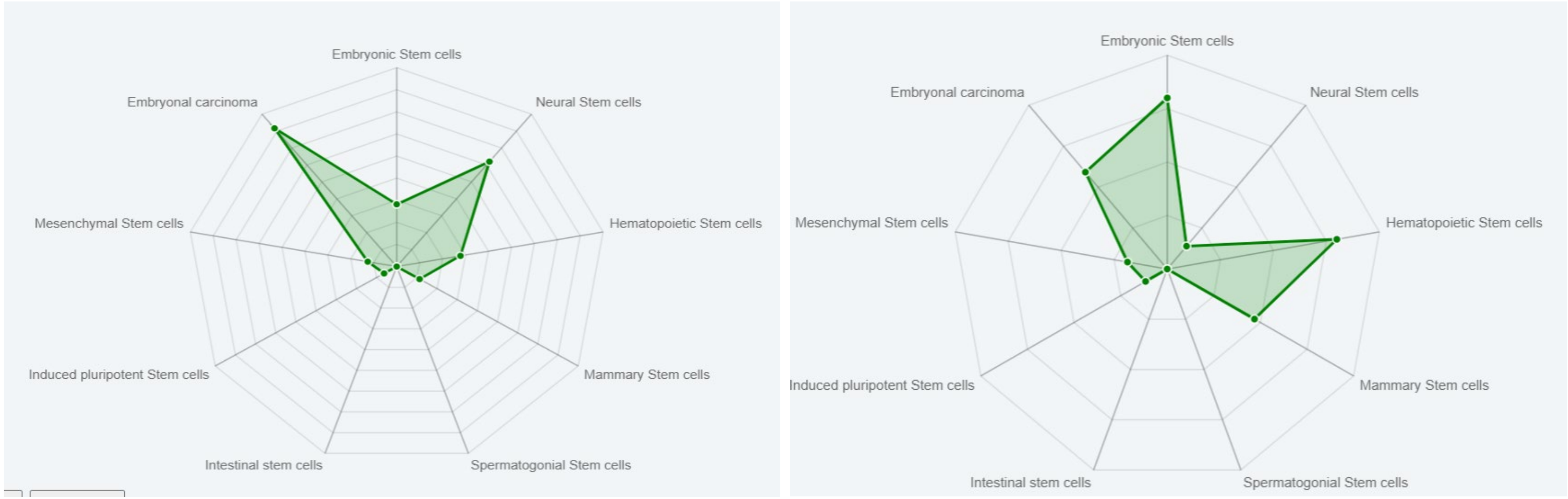

Figure S3. StemChecker (http://stemchecker.sysbiolab.eu/) was employed to evaluated *in-silic*o stemness signatures for LNCaP- and C4-2B- enzalutamide resistant cell lines represented in radar chart. This radar chart display highly significant enrichment for genes associated with embryonic stem cell type.

Supplemental Figure 4

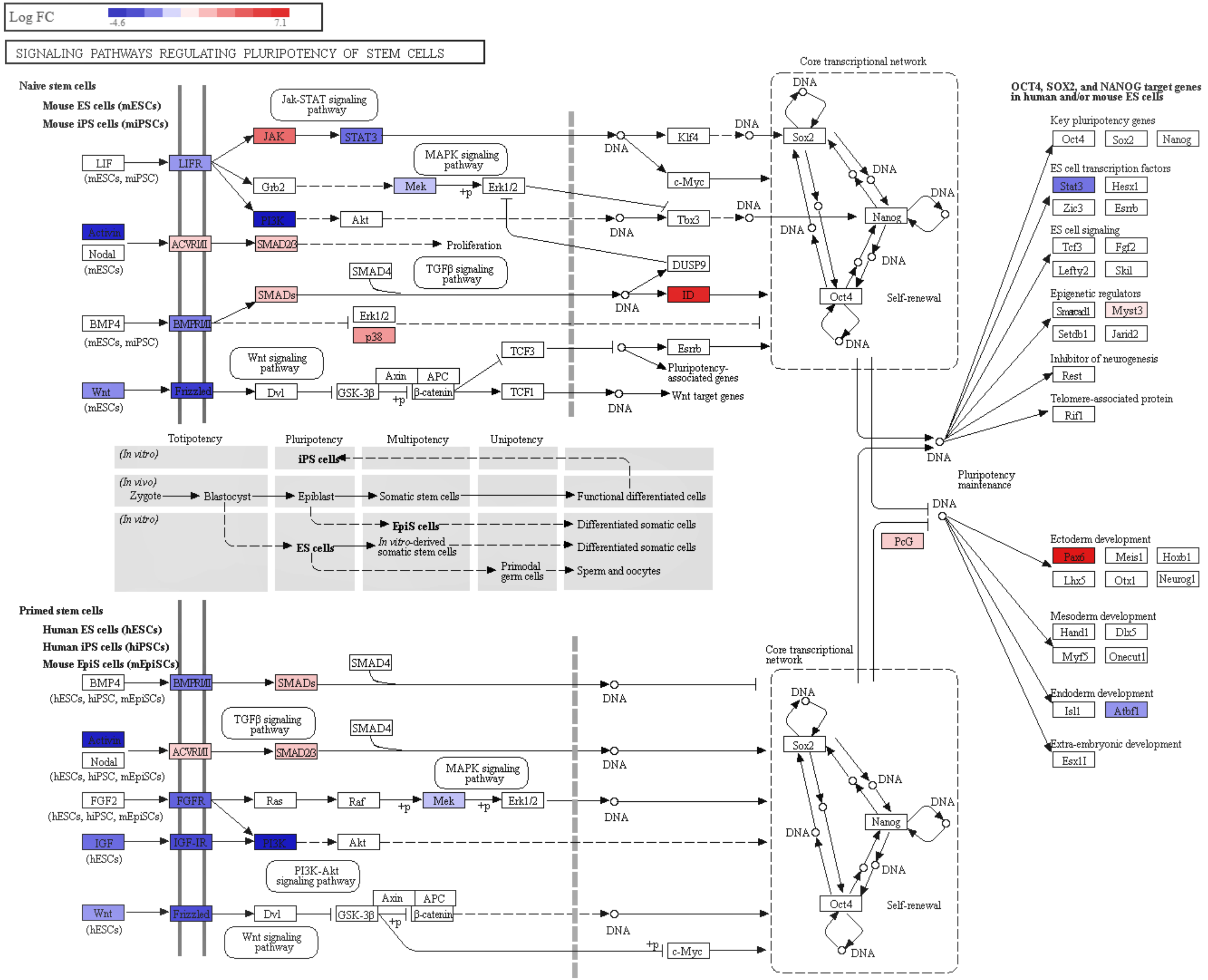

Figure S4. Signaling pathway regulating pluripotency of stem-cell (iPathway, Advaita) exhibit genes associated within and other allied signaling pathways (such as JAK-STAT signaling pathway) cooperatively leading to pluripotency maintenance.

Supplemental Figure 5

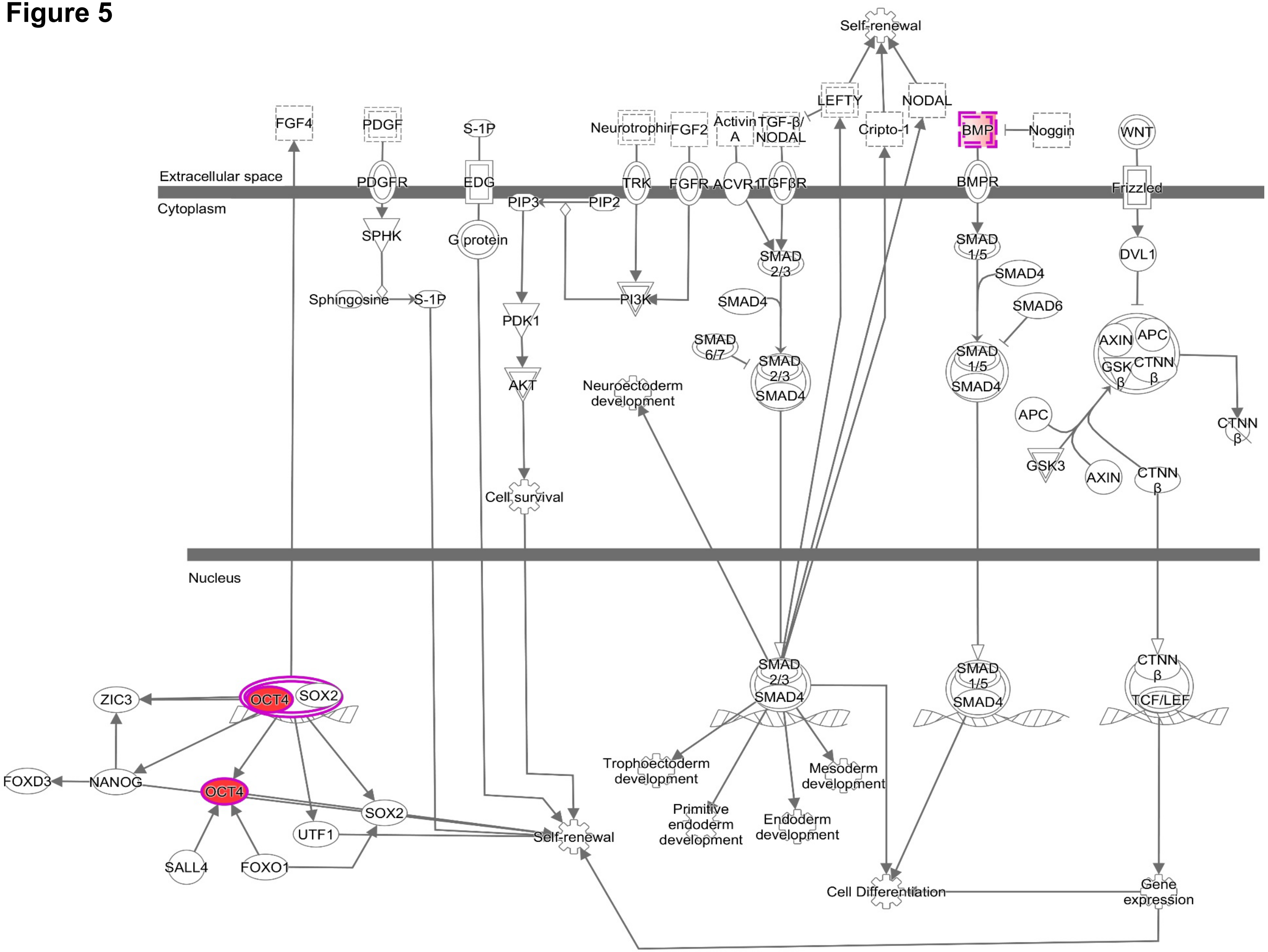

Figure S5. Human embryonic stem cell pluripotency signaling pathway (Ingenuity Pathway Analysis IPA), shows the orchestra between BMP signaling pathway and major transcription factors regulating pluripotency including, SOX2, OCT4, ALDH1 and NANOG. The role of OCT4/SOX2 is vital in the maintenance of cell pluripotency, and their function are reasoned essential for the process of self-renewal in human embryonic stem cells (ESCs). Thus human ESCs reveal a number of signaling pathways involved in self-renewal and pluripotency.
